# Association of Arts Event Attendance With Cognitive Function Among Older Adults Enrolled in the Health and Retirement Study

**DOI:** 10.1093/geroni/igad015

**Published:** 2023-03-11

**Authors:** John David Ike, Hwa Jung Choi, Tsai-Chin Cho, Joel D Howell, Kenneth M Langa

**Affiliations:** Department of Medicine, Duke University, Durham, North Carolina, USA; Trent Center for Bioethics, Humanities, & History of Medicine, Duke University, Durham, North Carolina, USA; Department of Internal Medicine, University of Michigan, Ann Arbor, Michigan, USA; Department of Health Management and Policy, University of Michigan, Ann Arbor, Michigan, USA; Department of Epidemiology, University of Michigan, Ann Arbor, Michigan, USA; Department of Internal Medicine, University of Michigan, Ann Arbor, Michigan, USA; Department of History, University of Michigan, Ann Arbor, Michigan, USA; Department of Internal Medicine, University of Michigan, Ann Arbor, Michigan, USA

**Keywords:** Arts engagement, Cognition, Community activity, Geriatrics, Humanities

## Abstract

**Background and Objectives:**

Among the cognitively impaired, arts engagement is associated with improved neurocognitive symptoms. Less is known about arts engagement as a potentially modifiable lifestyle factor to prevent or slow cognitive decline. Our aim was to evaluate the association between arts event attendance and cognition.

**Research Design and Methods:**

We used data from the 2014 and 2016 waves of the Health and Retirement Study to evaluate the association between arts event attendance and cognition using multivariable linear regressions. Arts event attendance in 2014 was our exposure of interest and included visiting an art museum or art gallery; attending an arts or crafts fair; attending a live performance (concert, play, or reading); and/or going to a movie theater. Cognitive function in 2016 measured on a 27-point scale by the Telephone Interview for Cognitive Status was our main outcome of interest.

**Results:**

Of the 1,149 participants included in the final analysis, 70.7% attended an arts event. The mean baseline cognitive score was higher among those who attended art events (16.8 [standard deviation {*SD*}, ±3.8] vs 13.8 [*SD*, ±5.0]; *p* < .001). In our multivariable regressions, those who attended arts events in 2014 exhibited higher cognitive scores in 2016 after controlling for demographic, socioeconomic, health, and baseline cognitive covariates (β, 1.07 [95% confidence interval {CI}, 0.50–1.64]; *p* < .001). This association was primarily observed in those with lower baseline cognitive function (β, 1.19 [95% CI, 0.33–2.06]; *p* = .008).

**Discussion and Implications:**

Arts event attendance may be associated with better cognitive function. Given concerns for residual confounding and reverse causality, this association warrants further study.

**Translational Significance:** There are limited pharmacologic treatments to slow or prevent cognitive decline. Recent studies have suggested that modifiable lifestyle factors may be associated with improved cognitive outcomes. Our study demonstrates that arts event attendance may be associated with improved cognitive function. This research may encourage additional exploration of nonpharmacologic lifestyle factors that are associated with improved cognition and healthy aging. This research will be of interest to various artists, social scientists, health care institutions, and cultural institutions who support investment in public arts-related infrastructure and programming to improve the health of their communities.

In the United States, 5–6 million adults are living with dementia, a geriatric syndrome characterized by cognitive impairment severe enough to interfere with independent function in daily life (Manly et al., 2022; Rajan et al., 2021). The number of U.S. adults with dementia is predicted to grow threefold by 2050 due to a large increase in the size of the older population (Plassman et al., 2007; Prince et al., 2013). Although many factors contribute to cognitive decline, we could prevent up to 30% of dementia cases by addressing modifiable risk factors such as cardiovascular diseases, education, physical activity, diet, and social engagement (Livingston et al., 2020; National Academies of Sciences et al., 2017; Plassman et al., 2010). Although pharmacologic agents garner national attention, other interventions warrant scientific exploration (Crosson et al., 2021; Dunn et al., 2021; Steinbrook, 2021). Arts engagement, including visiting museums and attending concerts, is a low-cost, nonpharmacologic, and scalable intervention that may positively affect cognitive function (Fancourt & Steptoe, 2018; Fancourt et al., 2018, 2021; Iizuka et al., 2019).

Among those with cognitive impairment, arts engagement is associated with improved attention, social behavior, self-esteem, short-term memory, and a decrease in neuropsychiatric symptoms (Chancellor et al., 2014; Iizuka et al., 2019; Mahendran et al., 2018; Noice & Noice, 2008; Noice et al., 2004; Parsa et al., 2010; Särkämö et al., 2014). Moreover, several programs including “Opening Minds through Art” and “Meet Me at MoMA” along with data from the National Endowment for the Arts further demonstrate the positive social and cognitive effects of arts engagement among those with cognitive impairment (Arts, 2013; Bienvenu & Hanna, 2017; Levenberg et al., 2021; Rajan & Rajan, 2017). The exact mechanism by which arts engagement improves cognition is unknown, but several theories have been suggested, including increased cognitive reserve and/or slowed neurodegeneration (Hultsch et al., 1999; Stern, 2002, 2012).

However, there remains considerable debate regarding art engagement’s ability to slow, delay, or prevent cognitive decline, and little is known about the variation in the potential effects of arts engagement on cognitive function. (Alain et al., 2019; Fancourt & Steptoe, 2018; Fancourt et al., 2018; Mahendran et al., 2018; McFadden & Basting, 2010; Rajan et al., 2018). Identifying subpopulations that may benefit cognitively from arts engagement could inform future interventions designed to improve cognitive function and/or delay cognitive impairments.

To examine the association between arts event attendance and cognition, we conducted a cohort study using data from the Health and Retirement Study (HRS), a nationally representative sample of older adults in the United States. We also assessed whether the potential effect of arts engagement on cognitive function differs by baseline cognitive function (high vs low function), among distinct demographic subpopulations, and if arts engagement is distinct from other forms of social engagement. We hypothesized that adults who attended arts events would exhibit better cognitive function after controlling for relevant demographic, socioeconomic, and health-related confounders. We also hypothesized that the association between arts event attendance and cognitive function would be more pronounced for those with low baseline cognitive function and that arts event attendance would be associated with improved cognitive function regardless of participation in other social engagement activities.

## Method

The study was exempt from IRB as the data are publicly available and do not contain identifying information. The Strengthening the Reporting of Observational Studies in Epidemiology (STROBE) criteria put forth by the Equator Network to increase uniformity and transparent reporting among cohort, case–control, and cross-sectional studies were followed (von Elm et al., 2007). The data were weighted to account for the differential probability of participant selection and adjusted for complex survey design. Data management and statistical analyses were performed using Stata (Stata Corp, Version 17.0; StataCorp, 2021). All analyses were two sided and a *p* value less than .05 was considered significant.

### Data and Sample

Established in 1992 and supported by the National Institute on Aging, the HRS is a longitudinal biennial survey that tracks the changing health, cognition, and economic circumstances of roughly 20,000 adults over the age of 51 living in the United States (HRS Staff, 2017; Sonnega et al., 2014). The HRS survey recruits new participants every 6 years based on a multistage area probability design, which utilizes geographic stratification and oversampling of particular demographic groups to maintain a representative cohort.

**Figure 1.**
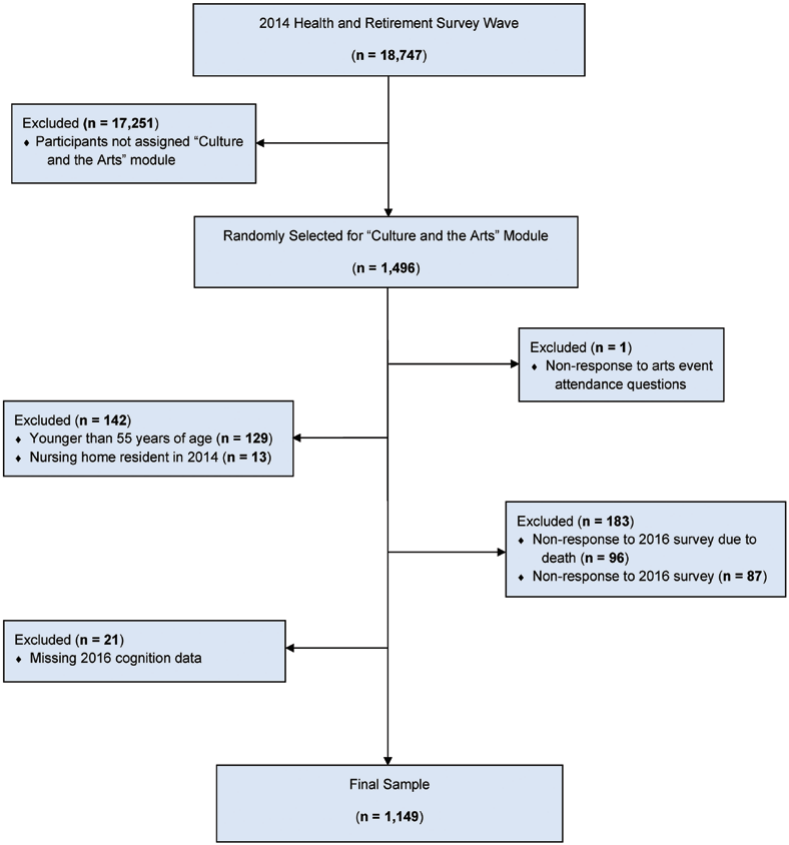
Study sample selection.

In 2014, the “Culture and the Arts” module was randomly assigned to 1,496 HRS participants to measure arts event attendance (the HRS surveyed 18,747 participants in 2014; Figure 1; HRS Staff, 2017; McFadden & Lunsman, 2009). From this subset of HRS participants, we first excluded those who lacked arts event attendance data (*n* =1). As our population of interest was community-dwelling older adults, we excluded participants younger than 55 years of age (*n* = 129) or nursing home residents in 2014 (*n* = 13). Because we assessed our outcome in 2016, we then excluded participants who lacked 2016 HRS survey data due to death (*n* = 96) or nonresponse (*n* = 87) followed by participants who had missing cognition data (*n* = 21). Our final sample size was 1,149.

### Outcome Variable—Cognitive Function

The HRS assesses cognitive function using measures adapted from the Telephone Interview for Cognitive Status (TICS) that correlates with formal in-person neuropsychiatric testing (Crimmins et al., 2011). This 27-point scale is the sum of subscores from the following assessment items: immediate recall test of 10 nouns (range 0–10), delayed recall of 10 nouns (range 0–10), a serial 7 subtraction test (range 0–5), and a backward count from 20 test (range 0–2; Crimmins et al., 2011). Higher scores indicate better cognitive function. The TICS can be administered in-person or by phone.

### Exposure Variables—Arts Event Attendance

Respondents were asked if they attended any of the following in the previous 12 months: an art museum or art gallery; an arts or crafts fair; a live performance (concert, play, or reading); and/or a movie screening in a theater. The survey also assessed the frequency of arts event attendance (more than once per week, once a week, 1–3 times a month, or less than once a month). To ensure sufficient power for analysis, a categorical arts event attendance variable was generated to include individuals who attended no arts events, individuals who attended arts events less often than once per month, and individuals who attended one or more arts event per month. We examined the frequency of arts event attendance as an exposure covariate to enable comparison to existing literature (Fancourt & Steptoe, 2018).

### Independent Covariates

We included multiple covariates to adjust for potential confounding. Demographic covariates were sex, age, self-reported race/ethnicity, and marital status. Race and ethnicities were categorized as Hispanic, non-Hispanic Black, non-Hispanic White, or non-Hispanic Other (American Indian, Alaskan Native, Asian, Native Hawaiian, Pacific Islander, and Other). Socioeconomic status covariates were net worth (in 2014 dollars) and education (<12 years, 12 years, 13–15 years, and ≥16 years). Self-reported chronic health conditions in the 2014 wave included: heart disease, stroke, hypertension, diabetes mellitus, arthritis, cancer, lung disease, and psychiatric illness.

### Leave Behind Questionnaire Covariates

To examine if the observed relationship between cognition and arts event attendance was influenced by, or distinct from, social interaction, we conducted a supplemental analysis using data from the 2014 HRS Psychosocial and Lifestyle Questionnaire (Leave Behind Questionnaire [LBQ]). Randomly assigned to 50% of participants who complete a face-to-face interview, the LBQ assesses the following social activities: volunteering with youth, attending educational or training courses, participating in nonreligious community organizations, participating in sports or social clubs, and performing other charity work (Smith et al., 2017). Of the 1,149 participants in our final sample, 488 completed the 2014 LBQ.

### Statistical Analysis

We first compared the demographic, socioeconomic, health, and cognitive characteristics of the entire 2014 HRS cohort to our final sample. Next, we examined the sample characteristics by the status of arts event attendance. We then performed *t* tests and chi-square tests to determine differences in estimates by arts event attendance status.

To assess the association between arts event attendance (2014) and cognitive function (2016), we used four multivariable linear regression models with sequential adjustments of 2014 baseline covariates: adjustment with baseline demographic covariates (sex, age, self-reported race/ethnicity, and marital status; Model 1); adjustment with Model 1 covariates plus socioeconomic covariates (net worth and education; Model 2); adjustment with Model 2 covariates plus health covariates (heart disease, stroke, hypertension, diabetes, arthritis, cancer, lung disease, and psychiatric illness; Model 3); and Model 3 covariates plus baseline cognition measured in 2014 (Model 4). We elected not to include baseline cognitive function in each model due to concerns for overcontrol and difficulty with result interpretation. Given the significant baseline cognitive differences between those who attended art events compared to those who did not attend events, we performed a stratified analysis by baseline cognitive score (2014) using a median split and repeated each linear regression model.

We also assessed the association between the frequency of arts event attendance (2014) and cognition (2016) using four sequential linear regression models after examining the demographic, socioeconomic, health, and cognitive differences between these three groups (did not attend, attended <1 event per month, and attended ≥1 event per month). Given significant differences in baseline cognition, we repeated each model using a median-split strategy by baseline cognitive score (2014).

To assess whether the relationship between arts event attendance and cognition is more pronounced in specific subpopulations, we performed stratified analyses by demographic and socioeconomic covariates using the most complete linear regression model (Model 4). Based on subgroup analysis results, we did not perform a moderating effect analysis, as our primary goal was to assess if the significant association between arts event attendance and cognition holds for all subgroups.

Finally, to check whether the potential relationship between arts event attendance and cognition was independent of other forms of social engagement, we analyzed the sample subset who completed the 2014 LBQ. First, we examined the proportion of participants who participated in each social activity before performing chi-square tests to determine the difference in social activity participation by arts event attendance status. We then assessed the association between arts event attendance (2014) and cognitive function (2016) using five multivariable linear regression models with sequential adjustment for demographic, socioeconomic, health, all 2014 LBQ social activity covariates, and cognition measured in 2014.

To ensure that the participants excluded due to nonresponse (*n* = 87), death (*n* = 96), or missing 2016 cognitive data (*n* = 21) did not have a significant impact on the results, auxiliary and sensitivity analyses were performed. First, we compared the baseline characteristics of the excluded sample versus our final sample. Second, we estimated the extent to which missing patterns (nonresponse, death, and missing 2016 cognitive data) differ by arts event attendance using two multinomial logistic regressions with the 2016 response status as the dependent variable and 2014 arts event attendance status as the independent variable. Third, we re-estimated the linear regression coefficient of 2016 cognition (dependent variable) by 2014 arts event attendance status (independent variable) based on different imputations of 2016 cognitive values ranging from the 10th, 25th, 50th, 75th, and 90th percentiles of the distribution.

## Results

### Sample Characteristics

The demographic, socioeconomic, health, and cognitive characteristics of those who were randomly selected to complete the “Culture and the Arts” module and *all* those surveyed in 2014 by the HRS were similar (see Supplementary Table 1). As summarized in Table 1, respondents who completed the “Culture and the Arts” module were predominantly female (58.0%), between the ages of 55 and 64 (49.0%), non-Hispanic White (77.0%), married or had a partner (65.1%), and, on average, attained at least a high school education (13.5 years of education [standard deviation {*SD*}, ±2.8 years]). Hypertension (60.7%) and arthritis (58.9%) were common. The mean cognitive score was 15.5 [*SD*, ±4.5] and the median cognitive score was 16.0 (interquartile range [IQR], 13.0–19.0).

**Table 1.** Baseline Demographic and Health Characteristics by Art Attendance Status (*N* = 1,149)

Characteristics	All participants	Attended arts event		*p* Value^*^
		Yes, *n* = 745 (70.7%)	No, *n* = 404 (29.3%)	
Female sex, *n* (%)	696 (58.0%)	464 (60.1%)	232 (52.8%)	.04
Age, *n* (%)				
55–64 years	494 (49.0%)	343 (53.6%)	151 (38.0%)	<.001
65–74 years	348 (32.0%)	229 (30.7%)	119 (35.1%)	
≥75 years	307 (18.9%)	173 (15.7%)	134 (26.9%)	
Age in years, Mean (*SD*)	66.6 (8.9)	65.6 (8.0)	69.1 (10.6)	<.001
Race/ethnicity, *n* (%)				
Black, non-Hispanic	239 (10.8%)	136 (9.4%)	103 (14.2%)	<.001
Hispanic	144 (8.5%)	65 (6.0%)	79 (14.5%)	
Other, non-Hispanic	36 (3.7%)	22 (3.4%)	14 (4.4%)	
White, non-Hispanic	730 (77.0%)	522 (81.3%)	208 (66.9%)	
Marital status, *n* (%)				
Never married	47 (5.1%)	23 (3.8%)	24 (8.2%)	.004
Married/partner	706 (65.1%)	486 (69.9%)	220 (53.5%)	
Separated/divorced	182 (15.3%)	112 (14.5%)	70 (17.3%)	
Widowed	213 (14.5%)	123 (11.7%)	90 (21.1%)	
Net worth (year 2014 $), *n* (%)				
Quartile 1 ($112,950–$50,000)	332 (23.2%)	152 (16.2%)	180 (40.1%)	<.001
Quartile 2 ($51,400–$209,000)	305 (25.4%)	182 (23.7%)	123 (29.4%)	
Quartile 3 ($210,000–$625,370)	273 (25.4%)	209 (28.9%)	64 (16.9%)	
Quartile 4 (≥$635,000)	239 (26.1%)	202 (31.2%)	37 (13.7%)	
Net worth, Mean (*SD*)	$585,331 ($1,286,301)	$697,066 ($1,384,182)	$315,756 ($777,216)	<.001
Net work (Median [IQR])	$226,000 ($60,000–$682,000)	$330,000 ($92,000–$797,000)	$94,000 ($5,000–$314,500)	<.001
Education, *n* (%)				
≤11 years	210 (12.9%)	74 (7.0%)	136 (27.1%)	<.001
12 years	333 (29.8%)	191 (26.5%)	142 (37.8%)	
13–15 years	301 (25.0%)	216 (26.1%)	85 (22.4%)	
≥16 years	299 (31.8%)	260 (40.0%)	39 (12.0%)	
Education in years, Mean (*SD*)	13.5 (2.8)	14.1 (2.3)	11.9 (3.4)	<.001
Health covariates, *n* (%)				
Heart disease	304 (25.0%)	186 (23.3%)	118 (29.3%)	.05
Stroke	79 (6.0%)	37 (4.0%)	42 (10.9%)	<.001
Hypertension	756 (60.7%)	461 (57.3%)	295 (69.1%)	<.001
Diabetes mellitus	298 (22.7%)	159 (18.4%)	139 (33.0%)	<.001
Arthritis	689 (58.9%)	431 (57.2%)	258 (63.1%)	.14
Cancer	176 (15.1%)	117 (15.1%)	59 (15.2%)	.96
Lung disease	117 (9.5%)	61 (8.4%)	56 (12.3%)	.15
Psychiatric Illness	221 (20.1%)	128 (18.0%)	93 (25.1%)	.03
Cognitive score (2014)				
Mean (*SD*)	15.9 (4.3)	16.8 (3.8)	13.8 (5.0)	<.001
Median (IQR)	16.0 (13.0–19.0)	17.0 (14.0–19.0)	14.0 (10.0–17.0)	<.001

*Notes*: Values in parentheses are weighted percentages derived from HRS sampling weights to adjust for complex survey design. HRS = Health and Retirement Study; IQR = interquartile range; *SD* = standard deviation.

^*^ Reported *p* values result from a chi-square or *t* test analysis where appropriate.

Among those who completed the “Culture and the Arts” module, 70.7% attended an arts event in the previous 12 months (Table 1). Compared to those who did not attend an arts event, those who did attend were more often female (60.1% vs 52.8%; *p* = .04), younger (65.6 years [*SD*, ±8.0 years] vs 69.1 years [*SD*, ±10.6 years]; *p* < .001), more likely to self-identify as a non-Hispanic White (81.3% vs 66.9%), more likely to be married or have a partner (69.9% vs 53.5%), wealthier (median net worth $330,000 [IQR, $92,000–$797,000] vs $94,000 [IQR, $5,000–$314,500]; *p* < .001), and more educated (14.1 years of education [*SD*, ±2.3 years] vs 11.9 years of education [*SD*, ±3.4 years]; *p* < .001). Individuals who attended art events were generally healthier, with a lower likelihood of heart disease (23.3% vs 29.3%; *p* = .05), stroke (4.0% vs 10.9%; *p* < .001), hypertension (57.3% vs 69.1%; *p* < .001), diabetes (18.4% vs 33.0%; *p* < .001), or psychiatric illness (18.0% vs 25.1%; *p* = .03). The median cognitive score among those who attended art events was, on average, higher than those who did not attend (17.0 [IQR, 14.0–19.0] vs 14.0 [IQR, 10.0–17.0]; *p* < .001).

Numerous demographic, socioeconomic, health, and cognitive differences were also noted among individuals who did not attend arts events compared to those who attended art events less often than once per month or greater than or equal to one time per month (Supplementary Table 2.

### Association Between Arts Event Attendance and Cognition

Table 2 reports the estimates from four sequential multivariable linear regression models in which the 2016 cognitive score is the dependent variable and 2014 arts event attendance is the independent variable. Model 1 shows that those who attended an arts event had, on average, 2.51-point higher cognitive scores in 2016 (cognitive score regression coefficient [β], 2.51 [95% confidence interval {CI}, 1.91–3.10]; *p* < .001) compared to those who did not attend an arts event when controlling for demographic characteristics (sex, age, race/ethnicity, and marital status). Additional adjustments for net worth and education (Model 2) and health covariates (Model 3) resulted in an additional decrease in 2016 cognitive score estimates (Model 2 β, 1.67 [95% CI, 1.05–2.28], *p* < .001; Model 3 β, 1.55 [95% CI, 0.95–2.16], *p* < .001). The inclusion of 2014 (baseline) cognition as an independent covariate (Model 4) had a modest effect on predicted 2016 cognitive scores estimates (β, 1.07 [95% CI, 0.50–1.64]; *p* < .001).

**Table 2.** Multivariable Linear Regression of 2016 Cognitive Scores by 2014 Arts Event Attendance (*N* = 1,149)

Regression models	Overall cognitive score regression coefficient (95% CI)	*p* Value	Cognitive score regression coefficient, by median-split 2014 cognitive scores (95% CI)			
			Low baseline cognitive score	*p* Value	High baseline cognitive score	*p* Value
Model 1 (demographics)	2.51 (1.91–3.10)	<.001	2.42 (1.70–3.14)	<.001	1.28 (0.41–2.16)	.005
Model 2 (demographics + socioeconomics)	1.67 (1.05–2.28)	<.001	1.80 (1.14–2.47)	<.001	0.83 (−0.06 to 1.72)	.07
Model 3 (demographics + socioeconomics + health)	1.55 (0.95–2.16)	<.001	1.52 (0.78–2.27)	<.001	0.78 (−0.10 to 1.66)	.08
Model 4 (demographics + socioeconomics + health + 2014 cognition)	1.07 (0.50–1.64)	<.001	1.19 (0.33–2.06)	.008	0.76 (−0.08 to1.60)	.07

*Notes*: Demographic covariates: age, sex, race/ethnicity, marital status; socioeconomic covariates: net worth, education; health covariates: heart disease, stroke, hypertension, diabetes mellitus, arthritis, cancer, lung disease, psychiatric illness. CI = confidence interval.

Splitting the cohort by the median cognitive score (2014) demonstrated that the association between arts event attendance and cognition remained significant among those with lower baseline cognitive scores in 2014 (Model 4 β, 1.19 [95% CI, 0.33–2.06]; *p* = .008) but was no longer significant among those with higher cognitive scores in our more adjusted models.

In a multivariable linear regression of 2016 cognitive scores by frequency of arts event attendance, we did not find a significant difference between those who attended less than one arts event per month (β, 1.08 [95% CI 0.51–1.65), *p* < .001) and those who attended greater than or equal to one arts event per month (β, 1.04 [95% CI, 0.27–1.81], *p* = .009) compared to those who did not attend when adjusting for demographic, socioeconomic, health, and 2014 cognitive covariates (Supplementary Table 3). Splitting the cohort by median cognitive score (2014) again demonstrated that only those with lower baseline cognitive scores had a significant association between arts event attendance and cognition; this association did not vary by frequency of arts event attendance (Supplementary Table 3).

### Arts Event Attendance and Cognitive Outcomes by Subgroups

**Figure 2.**
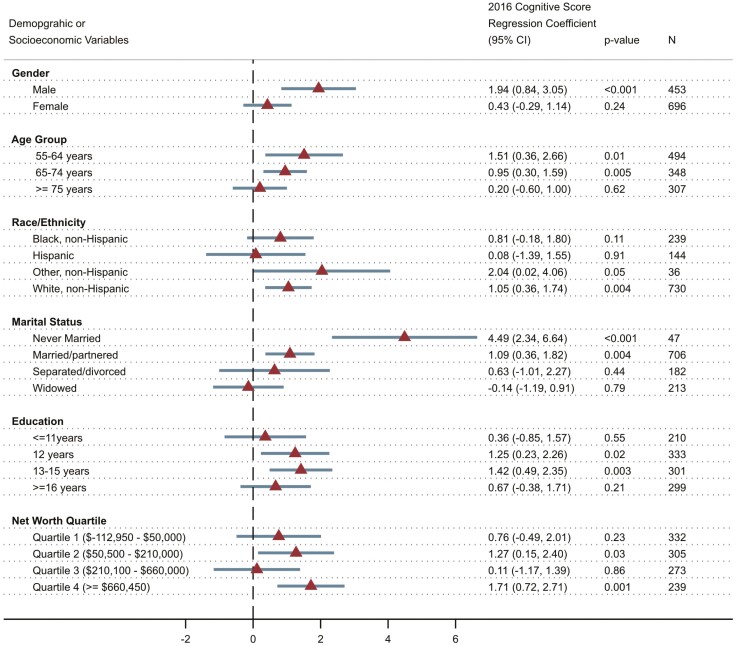
Association between art event attendance and cognition by subgroup.

Our stratified analysis suggests the relationship of arts event attendance to cognitive function held for several subgroups (Figure 2): males (β, 1.94 [95% CI, 0.84–3.05]; *p* < .001); individuals 55–64 years of age (β, 1.51 [95% CI, 0.36–2.66]; *p* = .01) and individuals 65–74 years of age (β, 0.95 [95% CI, 0.30–1.59]; *p* = .005); non-Hispanic other (β, 2.04 [95% CI, 0.02–4.06]; *p* = .05) and non-Hispanic Whites (β, 1.05 [95% CI, 0.36–1.74]; *p* = .004); individuals who were never married (β, 4.49 [95% CI, 2.34–6.64]; *p* < .001) and individuals who were married or partnered (β, 1.09 [95% CI, 0.36–1.82]; *p* = .004); individuals with 12 years of education (β, 1.25 [95% CI, 0.23–2.26]; *p* = .02) or 13–15 years of education (β, 1.42 [95% CI, 0.49–2.35]; *p* = .003); and those in wealth Quartile 2 (β, 1.27 [95% CI, 0.15–2.40]; *p* = .03) or wealth Quartile 4 (β, 1.71 [95% CI, 0.72–2.71]; *p* = .001).

### Analysis of Arts Event Attendance and Social Engagement

The results from our analysis of LBQ data are summarized in Table 3. In this subgroup of 488 participants, 73.8% attended an arts event, 36.7% volunteered with youth, 45.2% attended an educational or training course, 44.4% participated in a nonreligious community organization, 59.5% participated in a sports group or social club, and 53.6% engaged in charity work (Supplementary Table 4). In a bivariate analysis, those who attended art events were also more likely to engage in all social activities (*p* < .001; Supplementary Table 4).

**Table 3.** Multivariable Linear Regression of 2016 Cognitive Scores by 2014 Arts Event Attendance Among participants Who Completed the 2014 Leave Behind Questionnaire (*N* = 488)

Regression models	Cognitive score regression coefficient (95% CI)	*p* Value
Model 1 (demographics)	3.08 (2.26–3.91)	<.001
Model 2 (demographics + socioeconomics)	2.21 (1.27–3.15)	<.001
Model 3 (demographics + socioeconomics + health)	2.14 (1.23–3.05)	<.001
Model 4 (demographics + socioeconomics + health + social engagement covariates)	2.09 (1.15–3.03)	<.001
Model 5 (demographics + socioeconomics + health + social engagement covariates + 2014 cognition)	1.44 (0.54–2.35)	.002

*Notes*: Demographic covariates: age, sex, race/ethnicity, marital status; socioeconomic covariates: net worth, education. Health covariates: heart disease, stroke, hypertension, diabetes mellitus, arthritis, cancer, lung disease, psychiatric illness. Models 4 and 5 are adjusted for the following social activities: volunteering with youth, attending educational or training courses, participating in nonreligious community organizations, attending a sports or social club, and performing charity work. CI = confidence interval.

Our multivariable linear regression demonstrated that among participants who completed the 2014 LBQ, those who attended an arts event exhibited a 1.44-point higher cognitive score in 2016 compared to those who did not attend (Model 5 β 1.44 [95% CI, 0.54–2.35]; *p* = .002) after adjusting for demographic, socioeconomic, health, all social engagement covariates, and baseline cognition (Table 3).

### Auxiliary and Sensitivity Analysis—Examining Excluded Participants

The results of our auxiliary and sensitivity analyses are presented in Supplementary Tables 5–7. We observed numerous differences between those who were included in the final sample (*n* = 1,149), those who lacked 2016 cognition data (*n* = 21), those who were lost to follow up in 2016 (*n* = 87), and those who died (*n* = 96; Supplementary Table 5). These differences included demographic and socioeconomic characteristics (age, marital status, net worth, education), health differences (heart disease, stroke, diabetes, cancer), and baseline cognition (Supplementary Table 5). Results from an unadjusted multinomial logistic regression with 2016 response status as the dependent variable and arts event attendance as the independent variable suggests that compared to our final sample, those who attended art events in 2014 were 75% less likely to be missing cognition data in 2016 (*RRR* [relative risk reduction] 0.25 [95% CI 0.07–0.88]; *p* = .03) and 54% less likely to be dead in 2016 (*RRR* 0.46 [95% CI, 0.29–0.73]; *p* = .001; Supplementary Table 6). However, these differences were not significant after adjusting for demographic, socioeconomic, health, and 2014 cognitive covariates (Supplementary Table 6).

In multivariable linear regressions that included imputed 2016 cognitive scores for excluded participants (death, nonresponse, or missing 2016 cognition data), the association between arts event attendance and cognition remained (Supplementary Table 7). For example, if the 204 excluded participants possessed 2016 cognitive scores in the 50th percentile, those who attended arts events had, on average, 0.84-point higher cognitive scores in 2016 compared to those who did not attend (β 0.84 [95% CI, 0.34–1.34]; *p* = .001) after adjusting for demographic, socioeconomic, health, and baseline cognitive covariates.

## Discussion

Although our analyses could not fully address reverse causation or residual confounding, our results suggest that in a nationally representative sample of U.S. adults over the age of 55, individuals who attended arts events had, on average, 1.07-point higher cognitive scores on a 27-point scale 2 years later compared to those who did not attend. This finding persisted after controlling for multiple demographic, socioeconomic, health, and baseline cognitive covariates. The positive association between cognition and art attendance was independent of the frequency of attendance. However, our median-split stratified analyses demonstrated that these associations were only significant among those with lower baseline cognitive scores in 2014. Although the precise mechanism for this difference is unclear, it may be that people with lower baseline cognitive scores had more to gain from arts engagement.

Our findings support two prior observational studies from the United Kingdom, which suggested that arts event attendance was associated with a decreased incidence of dementia over a 10-year period and a lesser decline in cognitive function (Fancourt & Steptoe, 2018; Fancourt et al., 2018). Our research also suggests that the association between arts event attendance and cognition may be measurable on a 2-year time scale. Moreover, our outcome of interest (cognitive scores), as contrasted with a diagnosis of dementia, enables a more granular analysis of potential cognitive impacts. Interestingly, our analysis suggests that arts event attendance may only confer benefit to those with lower baseline cognitive functioning. Our study differs from some prior research in that we failed to find a dose relationship (Fancourt & Steptoe, 2018). However, the short follow-up time and small sample size limit our ability to make more specific conclusions about the effect of multiple exposures.

Some 30% of dementia cases may be preventable through the modulation of seven partially modifiable risk factors: diabetes, midlife hypertension, midlife obesity, physical inactivity, smoking, depression, and education attainment (Norton et al., 2014). Our study is consistent with these conclusions; we found that controlling for net worth, educational attainment, and health covariates accounted for nearly 52% of the difference in 2016 cognitive score estimates. Nevertheless, controlling for these and other potentially modifiable risk factors did not fully offset the significant association between attending arts events and higher cognitive scores.

Social engagement, in general, is associated with better cognition (Hofbauer & Rodriguez, 2021; James et al., 2011). Our analysis of 2014 LBQ data demonstrated a preserved association between attending arts events and higher cognitive scores even after controlling for other forms of social engagement (Table 3). This finding does not exclude the possibility that the social engagement often demanded from arts event attendance is somehow distinct from the other forms of socialization assessed in the LBQ.

Our auxiliary and sensitivity analyses suggest that despite numerous demographic, socioeconomic, health, and cognitive differences between our final sample and the excluded participants (death, nonresponse, or missing 2016 cognition data), the association between 2014 arts event attendance and 2016 cognition would remain if these participants were included at any imputed level of cognitive function. Additionally, supplementary analyses, which included baseline cognition in each model for all analyses (as opposed to the most adjusted models), modestly changed the strength of the association between arts event attendance and cognition, but not the significance.

How might arts event attendance improve cognition? One possible answer comes from cognitive reserve theory, the idea that a diversity of stimulating experiences offers protection against cognitive decline later in life through the development of neuroplasticity (Stern, 2002, 2012; Wu et al., 2016). For example, engaging the (traditionally) nondominant right hemisphere of the brain, which is frequently involved in arts engagement processes, may encourage neural connections that help to offset or delay cognitive decline. Similar to the theory of cognitive reserve, the “use it or lose it” hypothesis has also been proposed as a potential mechanism; neurodegeneration and cognitive decline accelerate in the absence of engaging stimuli, which may include the arts (Fancourt & Steptoe, 2018; Hultsch et al., 1999). Arts event attendance could also be categorized as a cognitive leisure activity, which is intellectually stimulating and may affect cognition (Iizuka et al., 2019).

In addition to the neurocognitive pathways that may be affected by arts event attendance, numerous programs targeting those with cognitive impairment or Alzheimer’s disease, including “Meet Me at MoMA,” demonstrate several positive externalities of arts engagement, including community building and emotional support (Bienvenu & Hanna, 2017; Parsa et al., 2010). Data from the National Endowment for the Arts also demonstrate improved quality of life, lower incidence of cardiovascular disease, and increased social connection among those who participate in arts-related events (Rajan & Rajan, 2017).

### Limitations

Several study limitations must be emphasized. First, the use of observational data limits our ability to identify causality and fully control for residual confounders. However, adjustments for demographic, socioeconomic, and health covariates controlled for many of the known associations with cognitive decline. Second, the “Culture and the Arts” module does not allow us to evaluate which type of arts event contributed to the observed association between arts event attendance and higher cognitive scores. Third, our cognitive outcome was obtained through the TICS (Crimmins et al., 2011). Although a validated measure, this approach is less precise than formal neuropsychiatric testing. Fourth, the health covariates utilized in our model are self-reported. Fifth, the HRS survey does not capture all cognitive leisure activities (e.g., crossword puzzles, board games) that have known associations with improved cognitive function (Iizuka et al., 2019). Lastly, there is the possibility of reverse causality, which we could not fully address in our analyses: Does arts event attendance lead to higher cognitive scores, or are those with higher cognitive scores more likely to attend arts events? This concern for reverse causality and confounding is further supported by the significant differences in baseline cognitive scores among those who attended arts events and those who did not attend. Regardless, our analysis supports prior observational studies in suggesting that community arts event attendance may be associated with better cognition.

## Conclusion

In a representative sample of U.S. adults, attending arts events was associated with better cognitive function. Given the potential threats to the validity of our findings (residual confounding, reverse causality), additional studies with longer follow-up periods are warranted. Should this finding be confirmed in future studies, it would suggest that arts event attendance may offer many health benefits to older adults, some of which could be cognitive. Moreover, this research could promote investment in public arts-related infrastructure and an increased incorporation of the arts into health care spaces which may help to mitigate the impending burden of cognitive impairment on patients, their families, and the U.S. health care system.

## Supplementary Material

igad015_suppl_Supplementary_Tables

## Data Availability

The data that support the findings of this study are available on request from the corresponding author. The data used in this study are publicly available through the Health and Retirement Study (https://hrs.isr.umich.edu/).
