# Supplementary material for: Association of Arts Event Attendance With Cognitive Function Among Older Adults Enrolled in the Health and Retirement Study
Source: Innov Aging. 2023 Mar 11;7(3):igad015. doi: 10.1093/geroni/igad015 (PMC10079814; doi:10.1093/geroni/igad015)
Supplement: igad015_suppl_Supplementary_Tables [file igad015_suppl_supplementary_tables.docx]

**Online Supplementary Material**

| **eTable 1. Baseline Demographic and Health Characteristics in the 2014 Health and Retirement Study and the "Culture and the Arts Module" Sample (N = 18,747)** | | | |
| --- | --- | --- | --- |
| **Characteristic, n (%)** | **2014 HRS Participants  (N = 18,747)** | **2014 "Culture and Arts" Sample (N = 1,149)** | **p-value^a^** |
| **Female Sex** | 11,048 (54.1%) | 696 (58.0%) | 0.02 |
| **Age** |  |  |  |
| < 55 years | 1561 (2.4%) | --- |  |
| 55-64 years | 6,802 (45.6%) | 494 (49.0%) | <0.001 |
| 65-74 years | 4,753 (30.1%) | 348 (32.0%) |  |
| => 75 years | 5,631 (21.9%) | 307 (18.9%) |  |
| Mean (SD), years | 67.0 (9.6) | 66.6 (8.9) | 0.12 |
| **Race/Ethnicity** |  |  |  |
| Black, non-Hispanic | 3,610 (10.0%) | 239 (10.8%) | 0.84 |
| Hispanic | 2,641 (8.8%) | 144 (8.5%) |  |
| Other, non-Hispanic | 652 (3.6%) | 36 (3.7%) |  |
| White, non-Hispanic | 11,815 (77.5%) | 730 (77.0%) |  |
| **Marital Status** |  |  |  |
| Never married | 903 (6.7%) | 47 (5.1%) | 0.35 |
| Married/Partner | 11,622 (64.4%) | 706 (65.1%) |  |
| Separated/Divorced | 2,620 (14.4%) | 182 (15.3%) |  |
| Widowed | 3,573 (14.4%) | 213 (14.5%) |  |
| **Net Worth (year 2014 $)** |  |  |  |
| Quartile 1 ($-112,950-$50,000) | 5,132 (25.0%) | 332 (23.2%) | 0.70 |
| Quartile 2 ($51,400-$209,000) | 4,506 (25.0%) | 305 (25.4%) |  |
| Quartile 3 ($210,000-$625,370) | 4,000 (25.1%) | 273 (25.4%) |  |
| Quartile 4 (>= $635,000) | 3,454 (24.9%) | 239 (26.1%) |  |
| Mean (SD) | $590,757 ($3,238,687) | $585,331 ($1,286,301) | 0.93 |
| Median (IQR) | $200,000 ($43,010-$598,000) | $226,000 ($60,000-$682,000) | 0.25 |
| **Education** |  |  |  |
| <=11 years | 3,878 (15.1%) | 210 (12.9%) | 0.30 |
| 12 years | 5,821 (29.8%) | 333 (29.8%) |  |
| 13-15 years | 4,515 (24.8%) | 301 (25.0%) |  |
| =>16 years | 4,446 (29.8%) | 299 (31.8%) |  |
| Mean (SD), years | 13.2 (3.0) | 13.5 (2.8) | 0.01 |
| **Health Covariates** |  |  |  |
| Heart Disease | 4,697 (23.8%) | 304 (25.0%) | 0.45 |
| Stroke | 1,530 (6.5%) | 79 (6.0%) | 0.72 |
| Hypertension | 11,672 (58.5%) | 756 (60.7%) | 0.29 |
| Diabetes Mellitus | 4,737 (22.7%) | 298 (22.7%) | 0.97 |
| Arthritis | 11,017 (57.6%) | 689 (58.9%) | 0.58 |
| Cancer | 2,901 (15.2%) | 176 (15.1%) | 0.97 |
| Lung disease | 1,982 (10.0) | 117 (9.5%) | 0.79 |
| Psychiatric Illness | 3,658 (19.5) | 221 (20.1%) | 0.89 |
| **Cognitive Score** |  |  |  |
| Mean (SD) | 15.9 (4.3) | 15.5 (4.5) | 0.48 |
| Median (IQR) | 16.0 (13.0-19.0) | 16.0 (13.0-19.0) | >0.99 |

*Notes.* Values in parentheses are weighted percentages derived from HRS sampling weights to adjust for complex survey design.

^a^ Reported p-values result from a chi-square or t test analysis where appropriate.

| **eTable 2. Baseline Demographic and Health Characteristics by Frequency of Arts Event Attendance (N = 1,149)** | | | | | |
| --- | --- | --- | --- | --- | --- |
| **Characteristic, n (%)** | **All Participants (N = 1,149)** | **Art Event Attendance Frequency** | | | **p-value^a^** |
|  |  | **Did Not Attend (N =406)** | **Attended < 1x /month (N = 476)** | **Attended >= 1x/month (N = 267)** |  |
|  |  |  |  |  |  |
| **Female Sex** | 696 (58.0%) | 234 (52.8%) | 300 (58.8%%) | 162 (62.8%) | 0.12 |
| **Age** |  |  |  |  |  |
| 55-64 years | 494 (49.0%) | 152 (38.0%) | 225 (55.7%) | 117 (49.5%) | < 0.001 |
| 65-74 years | 348 (32.0%) | 120 (35.1%) | 138 (29.2%) | 90 (33.6%) |  |
| => 75 years | 307 (18.9%) | 134 (26.8%) | 113 (15.0%) | 60 (16.9%) |  |
| Mean (SD), years | 66.6 (8.9) | 69.1 (10.6) | 65.3 (8.0) | 66.2 (8.1) | <0.001 |
| **Race/Ethnicity** |  |  |  |  |  |
| Black, non-Hispanic | 239 (10.8%) | 105 (14.3%) | 99 (10.9%) | 35 (6.3%) | 0.002 |
| Hispanic | 144 (8.5%) | 79 (14.5) | 40 (6.2%) | 25 (5.5%) |  |
| Other, non-Hispanic | 36 (3.7%) | 14 (4.4%) | 11 (2.9%) | 11 (4.4%) |  |
| White, non-Hispanic | 730 (77.0%) | 208 (66.8%) | 326 (80.0%) | 196 (83.7%) |  |
| **Marital Status** |  |  |  |  |  |
| Never married | 47 (5.1%) | 25 (8.2%) | 13 (2.8%) | 9 (5.7%) | 0.005 |
| Married/Partner | 706 (65.1%) | 220 (53.5%) | 305 (70.1%) | 181 (69.7%) |  |
| Separated/Divorced | 182 (15.3%) | 70 (17.2%) | 70 (14.1%) | 42 (15.3%) |  |
| Widowed | 213 (14.5%) | 91 (21.1%) | 87 (12.9%) | 35 (9.3%) |  |
| **Net Worth (year 2014 $)** |  |  |  |  |  |
| Quartile 1 ($-112,950-$50,000) | 332 (23.2%) | 181 (40.1%) | 95 (16.1%) | 56 (16.4%) | < 0.001 |
| Quartile 2 ($51,400-$209,000) | 305 (25.4%) | 124 (29.4%) | 137 (27.4%) | 44 (16.4%) |  |
| Quartile 3 ($210,000-$625,370) | 273 (25.4%) | 64 (16.9%) | 129 (28.4%) | 80 (29.9%) |  |
| Quartile 4 (>= $635,000) | 239 (26.1%) | 37 (13.7%) | 115 (28.2%) | 87 (37.2%) |  |
| Mean (SD) | $585,331 ($1,286,301) | $315,445 ($778,236) | $566,541 ($785,584) | $950,856 ($2,129,731) | < 0.001 |
| Median (IQR) | $226,000 ($60,000-$682,000) | $94,000 ($5,000-$314,500) | $272,500 ($92,000-$722,00) | $445,000 ($107,400-$1,060,000) | <0.001 |
| **Education** |  |  |  |  |  |
| <=11 years | 210 (12.9%) | 137 (27.1%) | 48 (7.2%) | 25 (6.7%) | <0.001 |
| 12 years | 333 (29.8%) | 142 (37.7%) | 143 (31.2%) | 48 (17.4%) |  |
| 13-15 years | 301 (25.0%) | 86 (22.4%) | 138 (27.3%) | 77 (23.8%) |  |
| =>16 years | 299 (31.8%) | 39 (12.0%) | 145 (34.1%) | 115 (51.5%) |  |
| Mean (SD), years | 13.5 (2.8) | 11.9 (3.4) | 13.9 (2.2) | 14.6 (2.5) | < 0.001 |
| **Health Covariates** |  |  |  |  |  |
| Heart Disease | 304 (25.0%) | 118 (29.3%) | 131 (25.2%) | 55 (19.5%) | 0.06 |
| Stroke | 79 (6.0%) | 42 (10.9%) | 24 (4.4%) | 13 (3.4%) | 0.002 |
| Hypertension | 756 (60.7%) | 296 (69.0%) | 298 (57.5%) | 162 (56.8%) | 0.005 |
| Diabetes Mellitus | 298 (22.7%) | 139 (33.0%) | 100 (18.1%) | 59 (19.0%) | <0.001 |
| Arthritis | 689 (58.9%) | 259 (63.1%) | 279 (56.7%) | 151 (58.0%) | 0.35 |
| Cancer | 176 (15.1%) | 59 (15.1%) | 74 (14.0%) | 43 (17.3%) | 0.53 |
| Lung disease | 117 (9.5%) | 56 (12.3%) | 42 (9.2%) | 19 (6.8%) | 0.17 |
| Psychiatric Illness | 221 (20.1%) | 94 (25.2%) | 79 (17.1%) | 48 (19.8%) | 0.08 |
| **Cognitive Score** |  |  |  |  |  |
| Mean (SD) | 15.5 (4.5) | 13.8 (5.0) | 16.7. (3.6) | 16.8 (4.0) | < 0.001 |
| Median (IQR) | 16.0 (13.0-19.0) | 14.0 (10.0-17.0) | 17.0 (14.0-19.0) | 17.0 (14.0-19.0) | <0.001 |

*Notes*. Values in parentheses are weighted percentages derived from HRS sampling weights to adjust for complex survey design.

^a^ Reported p-values result from a chi-square, Wald test, or t test analysis where appropriate.

| **eTable 3. Multivariable Linear Regression of 2016 Cognitive Scores by 2014 Arts Event Attendance Frequency (N = 1,149)** | | | | | | |
| --- | --- | --- | --- | --- | --- | --- |
| **Regression Models** | **Overall Cognitive Score Regression Coefficient (95% CI)** | **p-value** | **Cognitive Score Regression Coefficient, by median-split 2014 cognitive score (95% CI)** | | | |
|  |  |  | **Low baseline cognitive score** | **p-value** | **High baseline cognitive score** | **p-value** |
| **Model 1** (Demographics) | | | | | | |
| Did not attend | *ref* | | *ref* | | | |
| < 1x / month | 2.46 (1.85-3.07) | <0.001 | 2.44 (1.63-3.25) | < 0.001 | 1.21 (0.34-2.08) | 0.007 |
| => 1x / month | 2.60 (1.73-3.46) | <0.001 | 2.37 (1.31-3.44) | < 0.001 | 1.43 (0.36-2.49) | 0.01 |
| **Model 2** (Demographics + Socioeconomics) | | | | | | |
| Did not attend | *ref* | | *ref* | | | |
| < 1x / month | 1.72 (1.06-2.38) | <0.001 | 1.86 (1.08-2.64) | < 0.001 | 0.84 (-0.07-1.74) | 0.07 |
| => 1x / month | 1.54 (0.76-2.31) | <0.001 | 1.67 (0.68-2.65) | 0.001 | 0.82 (-0.19-1.84) | 0.11 |
| **Model 3** (Demographics + Socioeconomics + Health) | | | | | | |
| Did not attend | *ref* | | *ref* | | | |
| < 1x / month | 1.61 (0.96-2.27) | <0.001 | 1.57 (0.68-2.46) | 0.001 | 0.80 (-0.09-1.69) | 0.08 |
| => 1x / month | 1.41 (0.64-2.19) | 0.001 | 1.41 (0.45-2.37) | 0.005 | 0.74 (-0.27-1.74) | 0.15 |
| **Model 4** (Demographics + Socioeconomics + Health + 2014 Cognition) | | | | | | |
| Did not attend | *ref* | | *ref* | | | |
| < 1x / month | 1.08 (0.51-1.65) | <0.001 | 1.25 (0.24-2.26) | 0.02 | 0.77 (-0.07-1.62) | 0.07 |
| => 1x / month | 1.04 (0.27-1.81) | 0.009 | 1.08 (0.07 - 2.08) | 0.04 | 0.75 (-0.24-1.73) | 0.14 |

*Notes*. **Demographic covariates**: Age, Sex, Race/Ethnicity, Marital Status; **Socioeconomic covariates**: Net worth, Education; **Health covariates**: Heart Disease, Stroke, Hypertension, Diabetes Mellitus, Arthritis, Cancer, Lung Disease, Psychiatric Illness. 406 participants (29.4%) did not attend an arts event; 476 participants (46.6%) attended less than one arts event per month, and 267 (24.1%) participants attended greater than or equal to one arts event per month in the overall cohort.

| **eTable 4. Social and Art Engagement Activities among Participants who Completed the 2014 Leave Behind Questionnaire (N = 488)** | | | | |
| --- | --- | --- | --- | --- |
| **Social Activity, n (%)^a^** | **All Participants** n = 488 | **Attended Art Event** | | |
|  |  | **Yes** n = 324 (73.8%) | **No** n = 164 (26.2%) | **p-value^b^** |
| Attended Art Event | 324 (73.8%) | ---- | --- | --- |
| Volunteer with youth | 169 (36.7%) | 131 (43.5%) | 38 (17.4%) | < 0.001 |
| Education or training course | 193 (45.2%) | 156 (53.0%) | 37 (23.4%) | < 0.001 |
| Non-religious community organization | 204 (44.4%) | 171 (53.2%) | 33 (19.6%) | < 0.001 |
| Sports or social club | 273 (59.5%) | 213 (65.7%) | 60 (42.1%) | < 0.001 |
| Charity work | 252 (53.6%) | 196 (62.1%) | 56 (29.5%) | < 0.001 |

^a^ Values in parentheses are weighted percentages derived from HRS sampling weights to adjust for complex survey design.

^b^ Chi-square test

| **eTable 5. Baseline Demographic and Health Characteristics by 2016 Survey Status (N = 1,353)** | | | | | |
| --- | --- | --- | --- | --- | --- |
| **Characteristic, n (%)** | **Alive, Responded in 2016 N = 1,149 (86.8%)** | **Alive, Responded but Missing Cognition in 2016 N = 21 (1.2%)** | **Alive, Non-Response in 2016 N = 87 (5.9%)** | **Dead in 2016 N = 96 (6.1%)** | **p-value*** |
| **Female Sex** | 696 (58.0%) | 12 (56.0%) | 56 (66.6%) | 53 (53.1%) | 0.46 |
| **Age** |  |  |  |  |  |
| 55-64 years | 494 (49.0%) | 2 (19.4%) | 41 (38.7%) | 13 (16.1%) | < 0.001 |
| 65-74 years | 348 (32.0%) | 3 (11.1%) | 22 (37.4%) | 27 (30.5%) |  |
| => 75 years | 307 (18.9%) | 16 (69.6%) | 24 (23.8%) | 56 (53.4%) |  |
| Mean (SD), years | 66.6 (8.9) | 79.0 (14.5) | 68.5 (9.6) | 76.2 (10.9) | < 0.001 |
| **Race/Ethnicity** |  |  |  |  |  |
| Black, non-Hispanic | 239 (10.8%) | 3 (12.3%) | 16 (10.9%) | 16 (12.2%) | 0.40 |
| Hispanic | 144 (8.5%) | 5 (14.0%) | 12 (13.9%) | 5 (2.7%) |  |
| Other, non-Hispanic | 36 (3.7%) | 0.0 (0.0%) | 2 (1.7%) | 4 (4.8%) |  |
| White, non-Hispanic | 730 (77.0%) | 13 (73.7%) | 57 (73.5%) | 71 (80.4%) |  |
| **Marital Status** |  |  |  |  |  |
| Never married | 47 (5.1%) | 1 (4.7%) | 5 (8.6%) | 5 (8.2%) | 0.006 |
| Married/Partner | 706 (65.1%) | 10 (47.4%) | 46 (57.4%) | 42 (43.0%) |  |
| Separated/Divorced | 182 (15.3%) | 2 (5.6%) | 23 (16.0%) | 13 (17.0%) |  |
| Widowed | 213 (14.5%) | 8 (42.3%) | 13 (18.1%) | 36 (31.8%) |  |
| **Net Worth (year 2014 $)** |  |  |  |  |  |
| Quartile 1 ($-112,950-$50,000) | 332 (23.2%) | 10 (53.9%) | 34 (37.0%) | 33 (35.8%) | 0.01 |
| Quartile 2 ($51,400-$209,000) | 305 (25.4%) | 6 (22.2%) | 19 (18.1%) | 22 (23.0%) |  |
| Quartile 3 ($210,000-$625,370) | 273 (25.4%) | 4 (19.9%) | 22 (29.8%) | 23 (23.0%) |  |
| Quartile 4 (>= $635,000) | 239 (26.1%) | 1 (4.0%) | 12 (15.1%) | 18 (18.3%) |  |
| Mean (SD) | $585,331 ($1,286,301) | $215,743 ($608,795) | $309,670 ($473,051) | $436,976 ($1,026,397) | 0.01 |
| Median (IQR) | $226,000 ($60,000-$682,000) | $43,000 ($12,023-170,000) | $100,000 ($19,500-498,000) | $115,000 ($10,000-$496,000) | 0.02 |
| **Education** |  |  |  |  |  |
| <=11 years | 210 (12.9%) | 10 (47.9%) | 15 (21.9%) | 18 (17.1%) | 0.003 |
| 12 years | 333 (29.8%) | 5 (28.8%) | 32 (33.8%) | 39 (43.3%) |  |
| 13-15 years | 301 (25.0%) | 3 (14.0%) | 23 (24.9%) | 19 (16.5%) |  |
| =>16 years | 299 (31.8%) | 3 (9.4%) | 17 (19.4%) | 19 (22.7%) |  |
| Mean (SD), years | 13.5 (2.8) | 10.9 (3.5) | 12.6 (3.2) | 12.8 (2.7) | < 0.001 |
| **Health Covariates** |  |  |  |  |  |
| Heart Disease | 304 (25.0%) | 8 (46.6%) | 18 (21.5%) | 49 (50.6%) | < 0.001 |
| Stroke | 79 (6.0%) | 3 (16.0%) | 3 (2.4%) | 16 (14.1%) | 0.003 |
| Hypertension | 756 (60.7%) | 16 (78.4%) | 56 (63.7%) | 72 (72.7%) | 0.18 |
| Diabetes Mellitus | 298 (22.7%) | 10 (41.3%) | 19 (17.0%) | 40 (41.2%) | < 0.001 |
| Arthritis | 689 (58.9%) | 16 (68.4%) | 44 (58.6%) | 62 (68.0%) | 0.09 |
| Cancer | 176 (15.1%) | 5 (17.4%) | 6 (7.0%) | 36 (40.0%) | < 0.001 |
| Lung disease | 117 (9.5%) | 1 (7.0%) | 5 (8.1%) | 17 (16.9%) | 0.24 |
| Psychiatric Illness | 221 (20.1%) | 5 (24.3%) | 12 (15.0%) | 20 (23.2%) | 0.61 |
| **Cognitive Score** |  |  |  |  |  |
| Mean (SD) | 15.5 (4.5) | 9.8 (6.2) | 15.4 (4.5) | 12.4 (4.4) | < 0.001 |
| Median (IQR) | 16.0 (13.0-19.0) | 10.0 (5.0-14.0) | 16.0 (13.0-18.0) | 13.0 (10.0-15.0) | < 0.001 |

*Notes.* Values in parentheses are weighted percentages derived from HRS sampling weights to adjust for complex survey design.

^a^ Reported p-values result from a chi-square, Wald, or t test analysis where appropriate.

| **eTable 6. Multinomial Logistic Regression of 2016 Survey Response Status by 2014 Arts Event Attendance (N = 1,353)** | | |
| --- | --- | --- |
| **Regression Models** | **Relative Risk Reduction (95% CI)** | **p-value** |
| **Model 1 (**Unadjusted) | | |
| Alive, Responded | ***ref*** | |
| Alive, Missing Cognition Data | 0.25 (0.07-0.88) | 0.03 |
| Alive, Non-Response | 0.62 (0.34-1.15) | 0.13 |
| Died | 0.46 (0.29-0.73) | 0.001 |
| **Model 2** (Demographics + Socioeconomics + Health + 2014 Cognition) | | |
| Alive, Responded | ***ref*** | |
| Alive, Missing Cognition Data | 1.41 (0.48-4.20) | 0.53 |
| Alive, Non-Response | 0.77 (0.42-1.40) | 0.38 |
| Died | 1.08 (0.61-1.90) | 0.79 |

*Notes.* **Demographic covariates**: Age, Sex, Race/Ethnicity, Marital Status; **Socioeconomic covariates**: Net worth, Education; **Health covariates**: Heart Disease, Stroke, Hypertension, Diabetes Mellitus, Arthritis, Cancer, Lung Disease, Psychiatric Illness. Alive, responded (N = 1,149); Alive, missing cognition data (N = 21); Alive, Non-Response (N = 87); Died (N = 96).

| **eTable 7. Sensitivity Analysis - Multivariable Linear Regression of 2016 Cognitive Scores by 2014 Arts Event Attendance (N = 1,353)** | | |
| --- | --- | --- |
| **Regression Models^a,b^** | **Cognitive Score Regression Coefficient (95% CI)** | **p-value** |
| 10th percentile 2016 Cognitive Score (score=9) | 0.95 (0.41-1.49) | <0.001 |
| 25th percentile 2016 Cognitive Score (score=13) | 0.89 (0.40-1.37) | <0.001 |
| 50th percentile 2016 Cognitive Score (score=16) | 0.84 (0.34-1.34) | 0.001 |
| 75th percentile 2016 Cognitive Score(score=19) | 0.80 (0.24-1.36) | 0.01 |
| 90th percentile 2016 Cognitive Score (score=21) | 0.77 (0.15-1.38) | 0.02 |

^a^ Each model controlled for the following**: Demographic covariates**: Age, Sex, Race/Ethnicity, Marital Status; **Socioeconomic covariates**: Net worth, Education; **Health covariates**: Heart Disease, Stroke, Hypertension, Diabetes Mellitus, Arthritis, Cancer, Lung Disease, Psychiatric Illness; **2014 Cognitive Score**

^b^ 2016 Cognitive scores were imputed at the 10th, 25th, 50th, 75th, and 90th percentile for the following survey respondents: missing 2016 cognition data (N= 21); alive, but did not respond to 2016 survey (N=87); or died before 2016 survey (N= 96)
